# Association between the Functional Polymorphism of Vascular Endothelial Growth Factor Gene and Breast Cancer: A Meta-Analysis

**Published:** 2015-01

**Authors:** Juan Li, Yongjian Ju

**Affiliations:** Department of Radiotherapy, The Second Affiliated Hospital of Nantong University, Nantong University, Nantong 226001, Jiangsu Province, China

**Keywords:** Vascular endothelial growth factor A, Breast neoplasms, Polymorphism, Risk

## Abstract

The vascular endothelial growth factor (VEGF) gene single-nucleotide polymorphism involved in the regulation of the protein levels has been implicated in breast cancer. However, the published studies have produced contentious and controversial results. Herein, we performed a meta-analysis (from January to October 2013); to further evaluate the association between +936 C/T polymorphism and the risk of breast cancer. By searching the EMBASE, PubMed, and Web of Science databases, we identified a total of 12 case-control studies with 8,979 cancer patients and 9,180 healthy controls. The strength of the association was assessed using Odds Ratios (ORs) with 95% Confidence Intervals (CI). We found no evidence indicating that the allelic model or the genotype models of +936 C/T polymorphism were associated with the risk of breast cancer in total population (OR_CC vs. TT_=1.01, 95% CI=0.96-1.06, P_h_=1.00; OR_CC+CT vs. TT_=1.00, 95% CI=0.96-1.05, P_h_=1.00; OR_CC vs. CT+TT_=1.02, 95% CI=0.98-1.07, P_h_=0.94; OR _allele C vs. allele T_=1.01, 95% CI=0.98-1.04, P_h_=0.99; OR_CT vs. TT_=1.01, 95% CI=0.93-1.09, P_h_=1.00). Such lack of association with breast cancer was also observed in subgroup analyses according to ethnicity as well as in the analysis by source of controls. In conclusion, this meta-analysis suggests that the functionally important +936 C/T polymorphism may not be associated with breast cancer risk. Larger well-designed studies with gene-to-gene and gene-to-environment interactions are clearly required to validate the results further.

## Introduction

Angiogenesis is critical for the growth and metastasis of invasive tumors and constitutes an important component in the suppression of cancer formation.^1^ The process of transporting excess nutrients, producing some risk factors, and forming tumor blood vessels and a route for tumor cell egress induces tumor aggression, growth, and dissemination.^2^^,^^3^ Vascular endothelial growth factor (VEGF) acts as an angiogenic inducer that is an endothelial cell-specific mitogen, and as a mediator of vascular permeability, playing a central role in the regulation of this process. There has been much speculation that inhibition of VEGF activity may prevent tumor initiation, progression, and migration.^4^

Several lines of work have shown a significant involvement of VEGF in human carcinogenesis. Enhanced VEGF expression and increased intratumoral microvessel density are related to an advanced stage disease and worse prognosis for a variety of women-specific malignancies, such as ovarian cancer^5^^,^^6^ and breast cancer.^7^^,^^8^ The highly polymorphic VEGF gene comprises eight exons that produce different proteins by alternative splicing of a unique transcript generated from a single-copy gene.^9^ There are more than 100 single nucleotide polymorphisms (SNPs) identified in the VEGF gene to date (http://www.ncbi.nlm.nih.gov/SNP). A couple of polymorphisms within the region have been reported to be associated with the development of cutaneous malignant melanoma,^10^ lung cancer,^11^ and prostate cancer.^12^

The most extensively studied SNP has been a C-to-T transition in the 3’-untranslated region (+936 C/T). A connection between +936 C/T status and VEGF plasma levels has previously been established,^13^ and it is this relation that makes the polymorphism an ideal target for cancer research. A recent report indicated that +936 C/T polymorphism plays a major role in a broad range of human cancers, including breast cancer,^14^ a polygenic malignancy accounting for almost 16% of all cancer cases and 22.9% of female-specific diseases (http://www.who.int/cancer/detection/breast cancer/en/index1.html). However, current data on the association between VEGF +936 C/T and breast cancer susceptibility have shown a great discrepancy.^15^^-^^26^ Most importantly, the overall evidence of several meta-analyses, in which the included data are under intensive debate, suggested the association of +936 C/T polymorphism and breast cancer risk is not statistically significant.^27^^-^^31^ These findings seem to contradict the experimental evidence supplied in previous research. In the present study, we aimed to assess the debatable association further by means of meta-analysis based on corrected data and recently published studies to provide convincing evidence for the genetic contribution of +936 C/T genotypes in the risk of developing breast cancer.

## Materials and Methods

This meta-analysis was performed according to Moose Guidelines from January to October 2013 in Jiangsu Province (Nantong University), China. 

*Literature Search*

We carried out a systematic literature search for manuscripts reporting on +936 C/T polymorphism and the risk of breast cancer in EMBASE (http://www.embase.com/), PubMed (http://www.ncbi.nlm.nih.gov/pubmed), and Web of Science (http://newisiknowledge.com) without any language restriction, using a combination of the following search terms: “vascular endothelial growth factor” or “VEGF”, “+936 C/T” or “rs3025039”, “genotypes” or “variant” or “variants” or “polymorphism”or “polymorphisms”, and “breast cancer” (the last search updated in June 2013). We also hand-searched the reference lists cited in genetic association studies, narrative reviews and meta-analyses to further identify the relevant publications that may have missed in the electronic search. We additionally carried out hand searches of the journals known to publish SNP-breast cancer articles, including International Journal of Cancer, Medical Oncology, Breast Cancer Research, Cancer Letters, Clinical Cancer Research, Cancer Epidemiology, Biomarker & Prevention, Breast Cancer Research Treatment, et al. All titles, abstracts, and full-texts were carefully reviewed to check if there were usable data reported in the original articles. When the required data were absent, corresponding authors were contacted via e-mail. However, no response was received by this process. 

*Inclusion and Exclusion Criteria*

The following inclusion criteria were defined for the eligible studies in this meta-analysis: (a) the study must be published as a full-text paper with a case-control or cohort design; (b) the authors must have investigated the association between +936 C/T polymorphism and the risk of breast cancer; (c) the study must provide sufficient genetic data that could help to calculate an odds ratio (OR) and its 95% confidence intervals (CI); (d) the publication must be released online before the literature search was completed. We excluded the studies if; only breast cancer patients were investigated, information on genotype count was not detailed, or no responses were received from major authors. We also did not consider editorials, case reports, systematic reviews, and comment letters. In case of studies containing overlapped subjects, we selected the largest study with accessible genotype data.

*Data Extraction*

In order to maximize the reliability of data analyzed in this work, the data extraction was done by two independent reviewers (J Li and Yy Ding) based on a consensus reached on all items. The following characteristics were collected from each eligible article: first author’s surname, journal and year of publication, country where the study was carried out, ethnicity or racial descent (categorized as Caucasian or Asian), source of controls (categorized as population- or hospital-based study), baseline characteristics of the breast cancer patients and the disease-free individuals (e.g. mean age, smoking status, family history, body mass index, menopausal status, tumor size, regional lymph node metastasis, distant metastasis, histologic grade wherever available), assays utilized to genotype the polymorphism of interest, total cases and controls, genotype frequencies between cases and controls, matching status, adjusted factors and study design (categorized as retrospective or prospective). Accuracy of the extracted information was examined by crosscheck. Discrepancies, if any, were resolved through discussion with a senior reviewer (Yj Ju). 

*Quality Assessment*

The quality of included studies was assessed independently by the same two reviewers (J Li and Yy Ding) according to the P values of Hardy-Weinberg Equilibrium (HWE) in control groups.^32^ We defined the studies whose genotype distribution in controls was consistent with HWE (P>0.10) as high-quality studies, and those inconsistent with HWE as (P≤0.10) low-quality studies. We then conducted subgroup analyses according to quality (high quality, low quality) to confirm whether deviation from HWE had an influence on the association being investigated.

*Statistical Methods*

For the controls of each study, HWE was examined by the chi-square goodness-of-ﬁt test with P<0.05 representing a significant deviation. The OR and its 95% CI were calculated to assess the strength of the association between +936 C/T polymorphism and breast cancer risk. The pooled ORs were performed for allelic model: allele C vs. allele T, and genotype models: CC vs. TT (homozygote), CC+CT vs. TT (dominant), CC vs. CT+TT (recessive), and CT vs. TT (heterozygote). The Z test was applied to determine the statistical significance of summary ORs, and P<0.05 was deemed statistically significant. 

Heterogeneity assumption was evaluated by a chi-square based Q test. The significant level was defined at P<0.10. In addition, the I^2^ index indicating the proportion of inter-study variance attributable to heterogeneity rather than chance was used to measure the degree of inconsistency across studies. I^2^ statistics range from 0 and 100%, with higher proportions representing larger heterogeneity: 0-25% low heterogeneity, 25%-50% moderate heterogeneity, 50%-75% large heterogeneity, 75%-100% extremely large heterogeneity. The ﬁxed effect model derived from the Mantel-Haenszel method^33^ was used to pool the ORs when P>0.10 or I^2^ statistics less than 50%; otherwise, the random effect model described by Darlington^34^ was more appropriate. In addition, subgroup analyses according to ethnicity and source of controls were performed to detect the potential heterogeneity further.

The influence of each individual study on the summary ORs was evaluated by excluding the single studies in turn from the pooled estimate. Publication bias was examined visually by funnel-inverted plots in which the standard error of log (OR) of each study was plotted against its log (OR). Egger’s test was utilized to check the asymmetry of funnel plots on the natural logarithm scale of the ORs.^35^ An asymmetric plot and the P values of Egger’s linear regression test smaller than 0.10 suggested obvious publication bias.

All statistical data were analyzed with Stata software (Version 12.0, Stata Corporation, College Station, Texas, USA). All tests were two-sided with a statistically significant level at P<0.1. 

## Results

*Selection and Characteristics of Eligible Studies*

**Figure 1:**
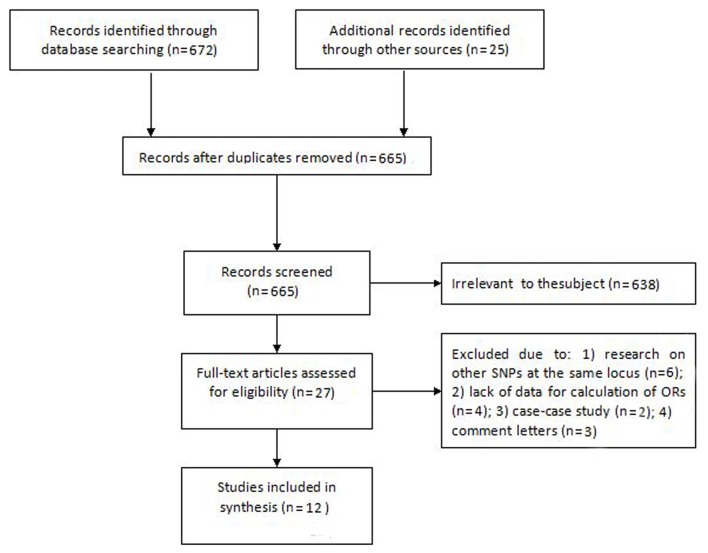
Moose chart showing selecting the final 12 studies included in this meta-analysis.

As depicted in figure 1, the computer-based search of EMBASE, PubMed, and Web of Science databases identified a total of 12 eligible studies^15^^-^^26^ for this meta-analysis, involving 8,979 breast cancer cases and 9,180 cancer-free controls. Although we primarily obtained 697 records, the initial removal of obviously irrelevant records and duplicates resulted in 27 studies for further evaluation. We subsequently deleted 15 studies due to various reasons, including research on other SNPs at the same locus, lack of data for calculation of ORs, case-case study, and comment letters.^36^^-^^50^ The main characteristics of the included studies are summarized in table 1. In terms of ethnicity, ten studies were based on Caucasian subjects and two on Asian subjects. The study by Jin et al. contained three sub-study groups from Poland, Germany, and Sweden respectively, and the three populations were taken as independent studies, which were merged into Caucasians in overall and subgroup meta-analysis. The quality of most included studies (83.3%) was high. Genotyping methods employed to determine VEGF SNP +936 C/T varied widely across studies, with the PCR-restriction fragment length polymorphism (PCR-RFLP) being most commonly used, followed by TaqMan and direct sequencing. The genotype distribution in controls of all studies was consistent with HWE except for the studies by Lin et al. and Luo et al.

**Table 1:** Main characteristics of all studies included in the meta-analysis

**First author**	**Year**	**Country **	**Ethnicity**	**Control source **	**Genotyping method**	**HWE**	**Quality **
Krippl^15^	2003	Austria	Caucasian	Population	PCR-RFLP	0.432	High
Jin^16^	2005	Sweden	Caucasian	Population	PCR-RFLP	0.940	High
Kataoka^17^	2006	China	Asian	Population	DS	0.129	High
Jacobs^18^	2006	USA	Caucasian	Population	TaqMan	0.602	High
Balasubramanian^19^	2007	UK	Caucasian	Population	PCR-RFLP	0.842	High
Pharoah^21^	2007	UK	Caucasian	Population	TaqMan	0.688	High
Eroglu^20^	2008	Turkey	Caucasian	Population	PCR-RFLP	0.843	High
Jakubowska^22^	2008	Poland	Caucasian	Population	PCR-RFLP	0.738	High
Lin^23^	2009	China	Caucasian	Hospital	PCR-RFLP	0.023	Low
Jakubowska^24^	2009	Poland	Caucasian	Population	PCR-RFLP	0.830	High
Oliveira^25^	2011	Brazil	Caucasian	Population	PCR-RFLP	0.201	High
Luo^26^	2013	China	Asian	Hospital	PCR-RFLP	0.004	Low

PCR: Polymerase chain reaction; PCR-RFLP: PCR-restriction fragment length polymorphism; DS: Direct sequencing; TaqMan: TaqManSNP; HWE: Hardy-Weinberg equilibrium

*Meta-Analysis Results*

**Figure 2:**
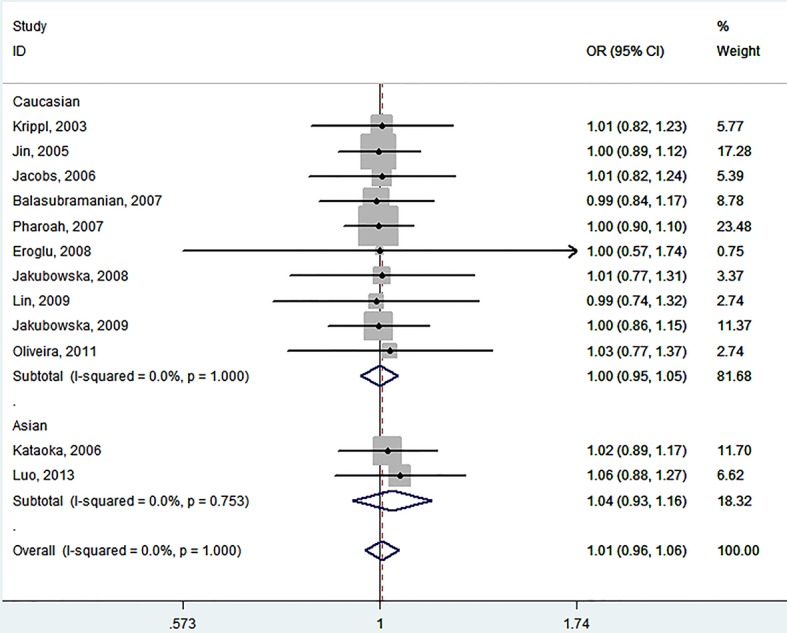
ORs of overall breast cancer risks associated with +936 C/T polymorphism under CC vs. TT model by ﬁxed effects for each of the 12 included studies. For each study, the estimates of OR and its 95% CI were plotted with a box and a horizontal line. ♦: Pooled OR and its 95% CI

**Figure 3:**
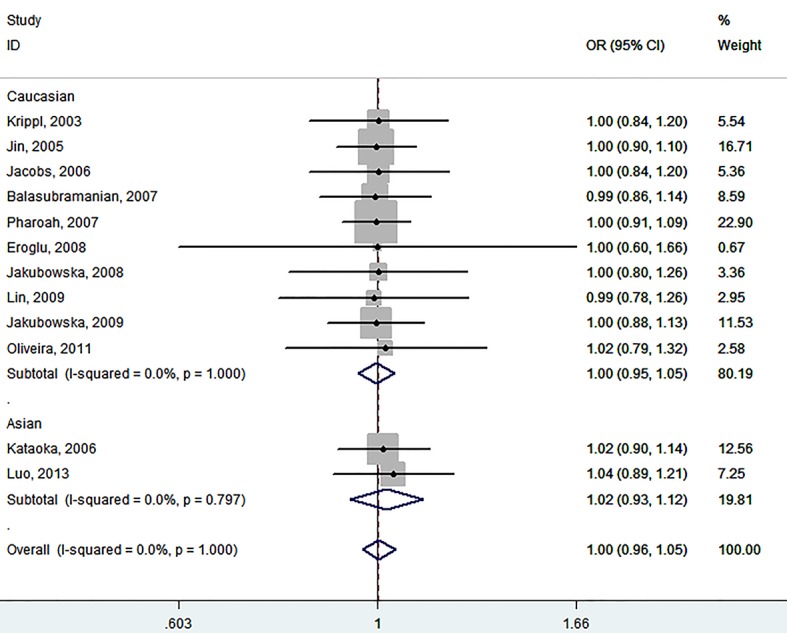
ORs of overall breast cancer risks associated with +936 C/T polymorphism under CC+CT vs. TT model by ﬁxed effects for each of the 12 included studies. For each study, the estimates of OR and its 95% CI were plotted with a box and a horizontal line. ♦: Pooled OR and its 95% CI

The main outcomes of this meta-analysis for +936 C/T polymorphism are listed in table 2. We found no evidence indicating that +936 C/T polymorphism were significantly associated with the risk of breast cancer in total population when using CC vs. TT (OR=1.01, 95% CI=0.96-1.06, P_h_=1.00). To test whether there is a positive association in another contrast models we subsequently assumed the CC+CT vs. TT model, failing to find any significant association (OR=1.00, 95% CI=0.96-1.05, P_h_=1.00). In terms of the remaining models tested, the statistical data showed no clear associations with the genetic risk of breast cancer (OR_CC vs. CT+TT_=1.02, 95% CI=0.98-1.07, P_h_=0.94; OR _allele C vs. allele T_=1.01, 95% CI=0.98-1.04, P_h_=0.99; OR_CT vs. TT_=1.01, 95% CI=0.93-1.09, P_h_=1.00) (see figure 2 and figure 3).

**Table 2:** Meta-analysis of the association between +936 C/T polymorphism and breast cancer risk

**Subgroups **	**CC vs. TT**		**CC+CT vs. TT**		**CC vs. CT+TT**		**C vs. T**		**CT vs. TT**	
	**OR (95% CI)**	** P_h_**	**OR (95% CI)**	** P_h_**	**OR (95% CI)**	** P_h_**	**OR (95% CI)**	** P_h_**	**OR (95% CI)**	** P_h_**
Ethnicity										
Caucasian	1.00 (0.95, 1.05)	1.00	1.00 (0.95, 1.05)	1.00	1.02 (0.97, 1.07)	0.87	1.01 (0.98, 1.04)	0.99	0.99 (0.90, 1.08)	1.00
Asian	1.04 (0.93, 1.16)	0.75	1.02 (0.93, 1.12)	0.79	1.02 (0.92, 1.13)	0.74	1.02 (0.96, 1.10)	0.68	1.07 (0.91, 1.26)	0.70
Control source										
Population	1.00 (0.95, 1.05)	1.00	1.00 (0.96, 1.05)	1.00	1.02 (0.97, 1.07)	0.90	1.01 (0.98, 1.04)	0.99	1.00 (0.92, 1.09)	1.00
Hospital	1.04 (0.89, 1.21)	0.69	1.03 (0.90, 1.17)	0.73	1.07 (0.92, 1.23)	0.69	1.04 (0.95, 1.15)	0.97	1.07 (0.86, 1.34)	0.54
Quality										
High-quality	1.00 (0.95, 1.05)	1.00	1.00 (0.96, 1.05)	1.00	1.02 (0.97, 1.07)	0.90	1.01 (0.98, 1.04)	0.99	1.00 (0.92, 1.09)	1.00
Low-quality	1.04 (0.89, 1.21)	0.69	1.03 (0.90, 1.17)	0.73	1.07 (0.92, 1.23)	0.69	1.04 (0.95, 1.15)	0.97	1.07 (0.86, 1.34)	0.54
All	1.01 (0.96, 1.06)	1.00	1.00 (0.96, 1.05)	1.00	1.02 (0.98, 1.07)	0.94	1.01 (0.98, 1.04)	0.99	1.01 (0.93, 1.09)	1.00

P_h_: P value of heterogeneity test; CI: Confidence interval; OR: Odds ratio

Since the general analysis, which included data from all published studies, only showed insignificant results, we thus performed a stratified analysis by ethnicity to estimate the risk for each subgroup. However, the negative results were not altered in the analysis of Caucasian populations: OR_CC vs. TT_=1.00, 95% CI=0.95-1.05, P_h_=1.0; OR_CC+CT vs. TT_=1.00, 95% CI=0.95-1.05, P_h_=1.0; OR_CC vs. CT+TT_=1.02, 95% CI=0.97-1.07, P_h_=0.87; OR _allele C vs. allele T_=1.01, 95% CI=0.98-1.04, P_h_=0.99; OR_CT vs. TT_=0.99, 95% CI=0.90-1.08, P_h_=1.0, and Asian populations OR_CC vs. TT_=1.04, 95% CI=0.93-1.16, P_h_=0.75; OR_CC + CT vs. TT_=1.02, 95% CI=0.93-1.12, P_h_=0.79; OR_CC vs. CT+TT_=1.02, 95% CI=0.92-1.13, P_h_=0.74; OR _allele C vs. allele T_=1.02, 95% CI=0.96-1.10, P_h_=0.68; OR_CT vs. TT_=1.07, 95% CI=0.91-1.26, P_h_=0.70. Similarly, the subgroups of hospital-based studies and population-based studies did not indicate any statistical evidence of significant breast cancer risk in relation to +936 C/T polymorphism at either the allelic or the genotypic level, nor did the final analysis according to the quality of study reveal a statistically significant association (see table 2).

*Sensitivity Analysis and Potential Bias*

**Figure 4:**
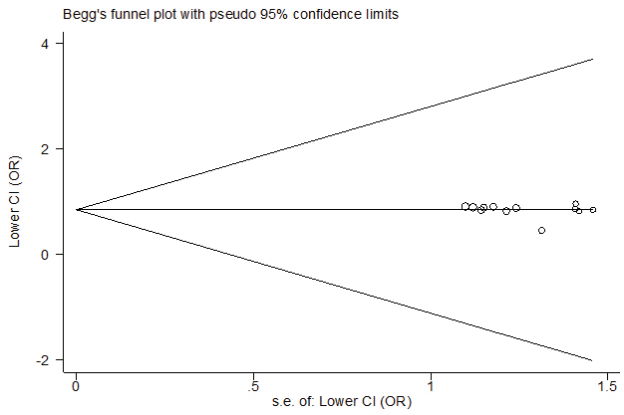
Funnel plot analysis to detect publication bias for +936 C/T polymorphism (CC vs. CT+TT)

Notable alternation in combined effect estimates is a common event when performing mete-analysis, where all genetic association studies addressing the same topic were incorporated. To check whether the pooled ORs were significantly affected by some specific study, we carried out a sensitivity analysis through sequential exclusion of each independent study. The data remained stable during the analysis and it is the unaltered ORs that ensure the reliability of our findings (data not shown). In the Begg’s test, no evidence of publication bias was detected by visual inspection of the funnel plots. P values of Egger’s test also indicated that there was no significant publication bias across the included studies (t=-0.70, P=0.49 for CC vs. CT+TT) (see figure 4).

## Discussion

Angiogenesis is a complicated process that could result in the formation of new blood vessels from pre-existing vasculature and thereby facilitates tumor growth, invasiveness, and development of metastasis. Data from experimental and clinical research have suggested that breast cancer is an angiogenesis-dependent disease closely associated with the serum level of VEGF.^51^ The potentially functional polymorphism in the VEGF gene, +936 C/T, was shown to correlate with lower VEGF production.^13^^,^^15^ It is the biological function of +936 C/T that leads to widespread attention towards the association between presence of +936 C/T polymorphism and breast cancer susceptibility. The published reports, however, have produced contentious and controversial results.^15^^-^^17^ Heterogeneous populations across the earlier association studies are a plausible explanation for the controversy. Other factors, such as varying study designs, number of individuals’ genotypes, different experimental methodologies may also contribute to the existing discrepancy among the studies.

Meta-analysis is an analytical tool that provides credible evidence for malignancy risk and well-characterized sequential variations by systematically summarizing existing data. In the current meta-analysis, we combined all available data on the association of +936 C/T polymorphism with breast cancer risk. The pooled analysis showed that there was no increased or decreased risk of breast cancer in correlation with any genotype or allele at +936 C/T. The lack of association with breast cancer was also observed in subgroup analyses according to ethnicity. In subsequent stratification analyses by source of controls and quality of study, nor did we find a clear association, the same as the previous analyses. 

Several meta-analyses have been carried out, in an attempt to identify the association of +936 C/T polymorphism with breast cancer risk. For instance, Gu et al.,^27^ who analyzed 5,729 cases and 5,868 controls, found +936 C/T polymorphism may not contribute to breast cancer susceptibility. This study was later commented and the association was re-evaluated in a recent publication, in which several methodological issues were addressed.^28^ The re-evaluation nevertheless failed to find any significant association. Consistent with the two earlier assessments, several meta-analyses reported in 2011 did not lend support modified breast cancer risk in relation to +936 C/T genotypes.^29^^-^^31^

One shared issue among these meta-analyses is that not all of the published data are included. In addition, there was substantial heterogeneity in each of the earlier meta-analyses, leading to an increased likelihood of biased results and hence making overall estimates less reliable. We enlarged our sample by incorporating all available data of the precious meta-analyses and the recently published studies, which helped to strengthen the statistical power and increased the precision of our findings, although we did not find any evidence of significant correlation. Another explanation may be that the polymorphism being investigated is a low-penetrance genetic variant and the minor impact on breast cancer susceptibility cannot be detected unless a sufficiently large analysis is performed. It is therefore worthwhile to determine the genetic association in a large-scale study. 

According to experimental evidence of functional genetic polymorphism at candidate locus, the VEGF +936T allele is speculated to have an association with predisposition to breast cancer. Activator protein 4 represents a helix-loop-helix transcription factor that upregulates serum levels of various cellular and viral genes through binding to particular sites, and it is these specific enhancer sites that modulate expression of VEGF.^52^^,^^53^ It has been shown that the +936T allele associated with reduced levels of VEGF abrogates activator protein 4 located in +936C allele, and thereby results in reduced VEGF expression.^13^ The +936T allele was also shown to have major effects on decreased uptake of 18F-fluorodeoxyglucose, a marker used to detect and diagnose breast cancer.^54^. It is therefore convincing that VEGF +936 C/T likely play a significant role in breast cancer susceptibility. If this speculation can be confirmed in the following studies, the +936 C/T polymorphism may be used as a biomarker for breast cancer.

We did not find a significant association between VEGF +936 C/T genotypes and breast cancer susceptibility in both Asian and Caucasian populations. The real association may be underestimated or even masked as a result of the small number of individuals in each ethnic group, the Asian group in particular (3, 664 subjects). The minor allele frequency (+936 C/T) of Asian populations (18.2%) is higher as compared to the global frequency (14.9%, http://www.ncbi.nlm.nih.gov/snp). Available data show a connection between the +936T allele and lower VEGF protein levels that is related to increased susceptibility of breast cancer, suggesting VEGF +936 C/T polymorphism may have effect modification of breast cancer in Asians. Our findings are obviously inconsistent with this hypothesis of a positive association and the inconsistency hints that the relation merits further investigation.

Several limitations of this study should be considered in interpreting our results. Firstly, mild deviation from HWE that probably results from methodological issues such as inappropriate or erroneous genotyping, selection bias and population categorization was detected in two studies,^23^^,^^26^ and this may have influenced our results, despite no indicated apparent alterations in the data with or without them. Secondly, we carried out an exhaustive literature search in medicine-specific databases and supplemental hand-search, failing to identify the publications written in other languages except English. As a result, only published data in the English language were analyzed in this meta-analysis. Further, unpublished data and ongoing studies were not considered in the present work, which may lead to biased results. Third, crude OR instead of adjusted OR was calculated to assess the risk of breast cancer. Since there was no uniform standard definition for the independent studies and the calculation of ORs varies substantially across the published studies. For example, Luo et al. adjusted ORs for sex and age, whereas the adjustment factors in the study by Oliveira et al. were age and race. The wide difference makes it impossible to provide an OR based on adjusted factors. Finally, it is known that breast cancer is an etiologically heterogeneous malignancy. Genetic contribution alone only accounts a part for the breast pathogenesis and the exact molecular mechanism that underlies this invasive disease remain largely unknown. Available data documented a significant involvement of gene-to-gene and gene-to-environment interactions in the development and progression of breast cancer, which, however, cannot be confirmed in this analysis due to lack of the original data.

The aforementioned shortcomings, nevertheless, could not overshadow the strong points of this quantitative assessment. We performed a meta-analysis with a maximum sample to date and it is the sample sufficiency that has contributed to robust and convincing findings. Besides, we updated earlier meta-analyses with new information from subsequent published studies and determined that +936 C/T genotypes were not involved in breast cancer incidence. Last but not least, publication bias and inter-study inconsistency (heterogeneity) are two major problems when performing meta-analysis. The confounding factors appeared to have no notable influence on this investigation, as suggested in the analytical methods.

In summary, the accumulated evidence from prospective studies supports that +936 C/T polymorphism is not significantly associated with the risk of breast cancer. Well-designed studies with a larger number of samples and with gene-to-gene and gene-to-environment interactions are considered necessary to clarify the association. 

This meta-analysis suggests that the common +936 C/T polymorphism may not be associated with breast cancer risk. This finding merits further research where the sample size is substantially large, and gene-gene and gene-environment interactions are considered. 

## References

[1] Folkman J (2002). Role of angiogenesis in tumor growth and metastasis. Semin Oncol.

[2] Makrilia N, Lappa T, Xyla V, Nikolaidis I, Syrigos K (2009). The role of angiogenesis in solid tumours: an overview. Eur J Intern Med.

[3] Kerbel RS (2008). Tumor angiogenesis. N Engl J Med.

[4] Ferrara N, Gerber HP (2001). The role of vascular endothelial growth factor in angiogenesis. Acta Haematol.

[5] Siddiqui GK, Elmasry K, Wong Te, Perrett C, Morris R, Crow JC (2010). Prognostic significance of intratumoral vascular endothelial growth factor as a marker of tumour angiogenesis in epithelial ovarian cancer. Eur J Gynaecol Oncol.

[6] Mabuchi S, Kawase C, Altomare DA, Morishige K, Hayashi M, Sawada K (2010). Vascular endothelial growth factor is a promising therapeutic target for the treatment of clear cell carcinoma of the ovary. Mol Cancer Ther.

[7] Poon RT, Fan ST, Wong J (2001). Clinical implications of circulating angiogenic factors in cancer patients. J Clin Oncol.

[8] Linderholm BK, Gruvberger-Saal S, Fernö M, Bendahl PO, Malmström P (2008). Vascular endothelial growth factor is a strong predictor of early distant recurrences in a prospective study of premenopausal women with lymph-node negative breast cancer. Breast.

[9] Wu BT, Su YH, Tsai MT, Wasserman SM, Topper JN, Yang RB (2004). A novel secreted, cell-surface glycoprotein containing multiple epidermal growth factor-like repeats and one CUB domain is highly expressed in primary osteoblasts and bones. J Biol Chem.

[10] Howell WM, Bateman AC, Turner SJ, Collins A, Theaker JM (2002). Influence of vascular endothelial growth factor single nucleotide polymorphisms on tumour development in cutaneous malignant melanoma. Genes Immun.

[11] Lee SJ, Lee SY, Jeon HS, Park SH, Jang JS, Lee GY (2005). Vascular endothelial growth factor gene polymorphisms and risk of primary lung cancer. Cancer Epidemiol Biomarkers Prev.

[12] Sfar S, Hassen E, Saad H, Mosbah F, Chouchane L (2006). Association of VEGF genetic polymorphisms with prostate carcinoma risk and clinical outcome. Cytokine.

[13] Renner W, Kotschan S, Hoffmann C, Obermayer-Pietsch B, Pilger E (2000). A common 936 C/T mutation in the gene for vascular endothelial growth factor is associated with vascular endothelial growth factor plasma levels. J Vasc Res.

[14] Eroglu A, Gulec S, Kurtman C, Cam R, Akar N (2006). Vascular endothelial growth factor 936 C/T polymorphism in cancer patients. Ann Oncol.

[15] Krippl P, Langsenlehner U, Renner W, Yazdani-Biuki B, Wolf G, Wascher TC (2003). A common 936 C/T gene polymorphism of vascular endothelial growth factor is associated with decreased breast cancer risk. Int J Cancer.

[16] Jin Q, Hemminki K, Enquist K, Lenner P, Grzybowska E, Klaes R (2005). Vascular endothelial growth factor polymorphisms in relation to breast cancer development and prognosis. Clin Cancer Res.

[17] Kataoka N, Cai Q, Wen W, Shu XO, Jin F, Gao YT (2006). Population-based case-control study of VEGF gene polymorphisms and breast cancer risk among Chinese women. Cancer Epidemiol Biomarkers Prev.

[18] Jacobs EJ, Feigelson HS, Bain EB, Brady KA, Rodriguez C, Stevens VL (2006). Polymorphisms in the vascular endothelial growth factor gene and breast cancer in the Cancer Prevention Study II cohort. Breast Cancer Res.

[19] Balasubramanian SP, Cox A, Cross SS, Higham SE, Brown NJ, Reed MW (2007). Influence of VEGF-A gene variation and protein levels in breast cancer susceptibility and severity. Int J Cancer.

[20] Eroğlu A, Oztürk A, Cam R, Akar N (2008). Vascular endothelial growth factor gene 936 C/T polymorphism in breast cancer patients. Med Oncol.

[21] Pharoah PD, Tyrer J, Dunning AM, Easton DF, Ponder BA, SEARCH Investigators (2007). Association between common variation in 120 candidate genes and breast cancer risk. PLoS Genet.

[22] Jakubowska A, Gronwald J, Menkiszak J, Górski B, Huzarski T, Byrski T (2008). The VEGF_936_C>T 3’UTR polymorphism reduces BRCA1-associated breast cancer risk in Polish women. Cancer Lett.

[23] Lin GT, Tseng HF, Yang CH, Hou MF, Chuang LY, Tai HT (2009). Combinational polymorphisms of seven CXCL12-related genes are protective against breast cancer in Taiwan. OMICS.

[24] Jakubowska A, Jaworska K, Cybulski C, Janicka A, Szymańska-Pasternak J, Lener M (2009). Do BRCA1 modifiers also affect the risk of breast cancer in non-carriers?. Eur J Cancer.

[25] Oliveira C, Lourenço GJ, Silva PM, Cardoso-Filho C, Favarelli MH, Gonçales NS (2011). Polymorphisms in the 5’- and 3’-untranslated region of the VEGF gene and sporadic breast cancer risk and clinicopathologic characteristics. Tumour Biol.

[26] Luo T, Chen L, He P, Hu QC, Zhong XR, Sun Y (2013). Vascular endothelial growth factor (VEGF) gene polymorphisms and breast cancer risk in a chinese population. Asian Pac J Cancer Prev.

[27] Gu D, Wang M (2011). VEGF 936C>T polymorphism and breast cancer risk: evidence from 5,729 cases and 5,868 controls. Breast Cancer Res Treat.

[28] Jin B, Jiang F, Ding Z (2011). Revaluation of the association between vascular endothelial growth factor gene 936 C/T polymorphism and breast cancer risk. Breast Cancer Res Treat.

[29] Qiu LX, Wang K, Yang S, Mao C, Zhao L, Yao L (2011). Current evidences on vascular endothelial growth factor polymorphisms and breast cancer susceptibility. Mol Biol Rep.

[30] Wang K, Liu L, Zhu ZM, Shao JH, Xin L (2011). Five polymorphisms of vascular endothelial growth factor (VEGF) and risk of breast cancer: a meta-analysis involving 16,703 individuals. Cytokine.

[31] Yang DS, Park KH, Woo OH, Woo SU, Kim AR, Lee ES (2011). Association of a vascular endothelial growth factor gene 936 C/T polymorphism with breast cancer risk: a meta-analysis. Breast Cancer Res Treat.

[32] Salanti G, Amountza G, Ntzani EE, Ioannidis JP (2005). Hardy-Weinberg equilibrium in genetic association studies: an empirical evaluation of reporting, deviations, and power. Eur J Hum Genet.

[33] Altman DG, Royston P (2000). What do we mean by validating a prognostic model?. Stat Med.

[34] Darlington GA, Donner A (2007). Meta-analysis of community-based cluster randomization trials with binary outcomes. Clin Trials.

[35] Schwarzer G, Antes G, Schumacher M (2007). A test for publication bias in meta-analysis with sparse binary data. Stat Med.

[36] James R, Ramesh G, Krishnamoorthy L, Bhagat R, Chadaga S, Deshmane V (2014). Prevalence of +405G>C,-1154G>A Vascular Endothelial Growth Factor Polymorphism in Breast Cancer. Indian J Clin Biochem.

[37] Liu L, Hua FZ, Cao JQ, Zhu PQ (2011). VEGF 936C>T polymorphism and breast cancer risk: evidence needed further clarification. Breast Cancer Res Treat.

[38] Liu S, Qian W (2012). Need for clarification of data in a recent meta-analysis on vascular endothelial growth factor (VEGF) and risk of breast cancer. Cytokine.

[39] Clar H, Krippl P, Renner W, Langsenlehner T, Clar V, Windhager R (2009). Association of polymorphisms of angiogenesis genes with breast cancer. Breast Cancer Res Treat.

[40] Sa-Nguanraksa D, O-Charoenrat P (2012). The role of vascular endothelial growth factor a polymorphisms in breast cancer. Int J Mol Sci.

[41] Etienne-Grimaldi MC, Formento P, Degeorges A, Pierga JY, Delva R, Pivot X (2011). Prospective analysis of the impact of VEGF-A gene polymorphisms on the pharmacodynamics of bevacizumab-based therapy in metastatic breast cancer patients. Br J Clin Pharmacol.

[42] Kidd LR, Brock GN, VanCleave TT, Benford ML, Lavender NA, Kruer TL (2010). Angiogenesis-associated sequence variants relative to breast cancer recurrence and survival. Cancer Causes Control.

[43] Keefe SM, Demichele A (2010). The expanding role of bevacizumab in the treatment of human epidermal growth factor receptor 2-negative breast cancer. Curr Oncol Rep.

[44] Knechtel G, Hofmann G, Gerger A, Renner W, Langsenlehner T, Szkandera J (2010). Analysis of common germline polymorphisms as prognostic factors in patients with lymph node-positive breast cancer. J Cancer Res Clin Oncol.

[45] Jain L, Vargo CA, Danesi R, Sissung TM, Price DK, Venzon D (2009). The role of vascular endothelial growth factor SNPs as predictive and prognostic markers for major solid tumors. Mol Cancer Ther.

[46] Wehrschuetz M, Schöllnast H, Wehrschuetz E, Renner W, Luschin G (2009). VEGF 936C > T Polymorphism and Association of BI-RADS Score in Women with Suspected Breast Cancer. Breast Cancer (Auckl).

[47] Schneider BP, Radovich M, Sledge GW, Robarge JD, Li L, Storniolo AM (2008). Association of polymorphisms of angiogenesis genes with breast cancer. Breast Cancer Res Treat.

[48] Langsenlehner U, Wolf G, Langsenlehner T, Gerger A, Hofmann G, Clar H (2008). Genetic polymorphisms in the vascular endothelial growth factor gene and breast cancer risk. The Austrian “tumor of breast tissue: incidence, genetics, and environmental risk factors” study. Breast Cancer Res Treat.

[49] Lu H, Shu XO, Cui Y, Kataoka N, Wen W, Cai Q (2005). Association of genetic polymorphisms in the VEGF gene with breast cancer survival. Cancer Res.

[50] Smith KC, Bateman AC, Fussell HM, Howell WM (2004). Cytokine gene polymorphisms and breast cancer susceptibility and prognosis. Eur J Immunogenet.

[51] Morabito A, Sarmiento R, Bonginelli P, Gasparini G (2004). Antiangiogenic strategies, compounds, and early clinical results in breast cancer. Crit Rev Oncol Hematol.

[52] Keerthivasan S, Keerthivasan G, Mittal S, Chauhan SS (2007). Transcriptional upregulation of human cathepsin L by VEGF in glioblastoma cells. Gene.

[53] Cao J, Tang M, Li WL, Xie J, Du H, Tang WB (2009). Upregulation of activator protein-4 in human colorectal cancer with metastasis. Int J Surg Pathol.

[54] Wolf G, Aigner RM, Schaffler G, Langsenlehner U, Renner W, Samonigg H (2004). The 936C>T polymorphism of the gene for vascular endothelial growth factor is associated with 18F-fluorodeoxyglucose uptake. Breast Cancer Res Treat.

